# Fishing for Trouble: A Novel Surgical Technique for Penetrating Fishhook Injuries of the Eyelid

**DOI:** 10.7759/cureus.36478

**Published:** 2023-03-21

**Authors:** Nathan J Brown, Alex T Legocki, Emily K Tam, Francine M Baran

**Affiliations:** 1 Ophthalmology, Washington State University Elson S. Floyd College of Medicine, Spokane, USA; 2 Ophthalmology, University of Washington, Seattle, USA; 3 Ophthalmology, Seattle Children's Hospital, Seattle, USA

**Keywords:** clamp and retract, removal technique, eyelid foreign body, fishhook injury, fishhook removal

## Abstract

Fishhook injuries commonly occur and may present as ophthalmic surgical emergencies. Choosing the appropriate removal technique is critical and depends on the involved extra- and intra-ocular structures and hook characteristics. We describe the case of a challenging fishhook removal where a novel surgical technique was developed. An eight-year-old boy presented with a full-thickness fishhook injury to the eyelid. During removal surgery, the thickness and density of the fishhook prevented surgical tools from transecting the shank. A novel approach was deemed necessary for safe removal, termed the clamp and retract technique. To our knowledge, this is its first documented use in the literature.

## Introduction

Angling has been practiced for hundreds of years and continues to be a popular tradition in sports, the commercial industry, and cultural practices around the globe. Modern-day lures commonly consist of one to three branching shanks with either a barbed or barbless hook. Fishhook injuries commonly occur and, in some cases, may present as ophthalmologic emergencies. Thus, prompt evaluation of extra- and intra-ocular structures and identification of hook characteristics are critical for determining the appropriate removal technique.

Penetrating globe injuries and removal techniques have been well documented in the literature [1-3]. However, there are few reports of fishhook eyelid injuries, especially in pediatric cases. We discuss a modified removal technique for a full-thickness fishhook injury of the eyelid, termed the advance and clamp technique. As described in our case, the thickness and density of the fishhook prevented surgical tools from safely transecting the shank. Our novel technique safely removed a thick-barbed fishhook from the left lower eyelid without complications, where previously documented techniques were unsuccessful. To our knowledge, this is its first documented use in the literature.

## Case presentation

**Figure 1:**
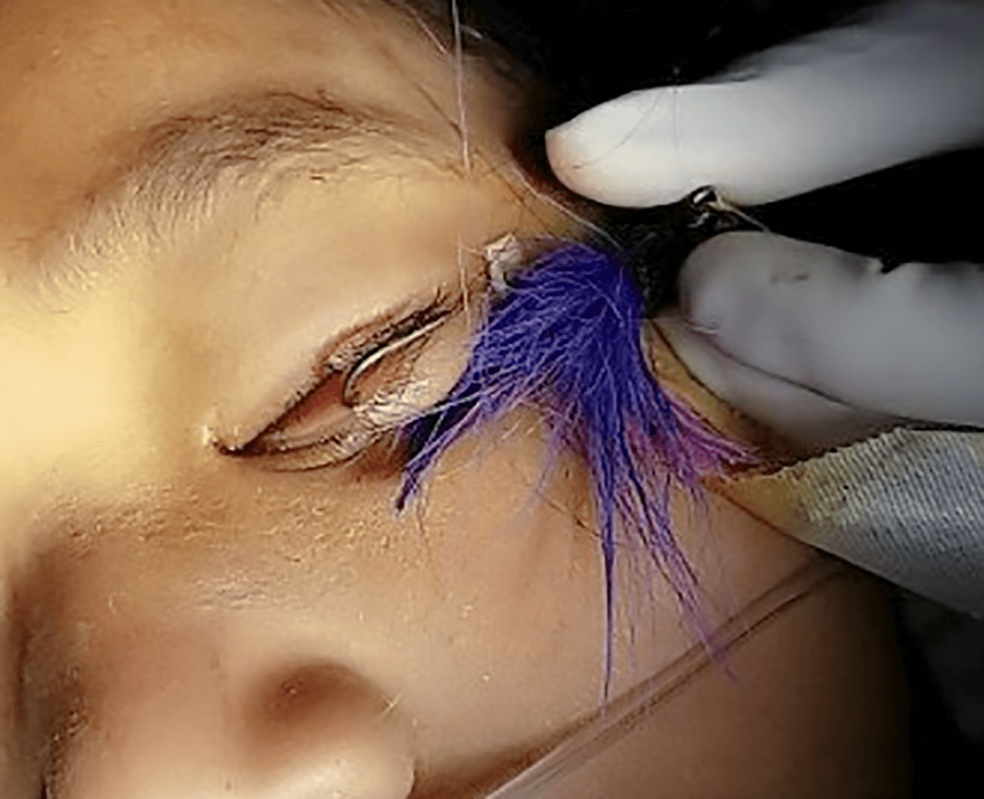
Penetrating fishhook injury connecting full thickness to a 1mm wound just internal to the left lower lid margin.

The patient was an 8-year-old boy who presented with a single-barbed fishhook embedded in his left lower eyelid (Figure 1). Initial physical examination was limited due to discomfort and cooperation. He was recommended for examination under general masked anesthesia, removal of a left lower lid foreign body, and possible left lower eyelid repair.

**Figure 2:**
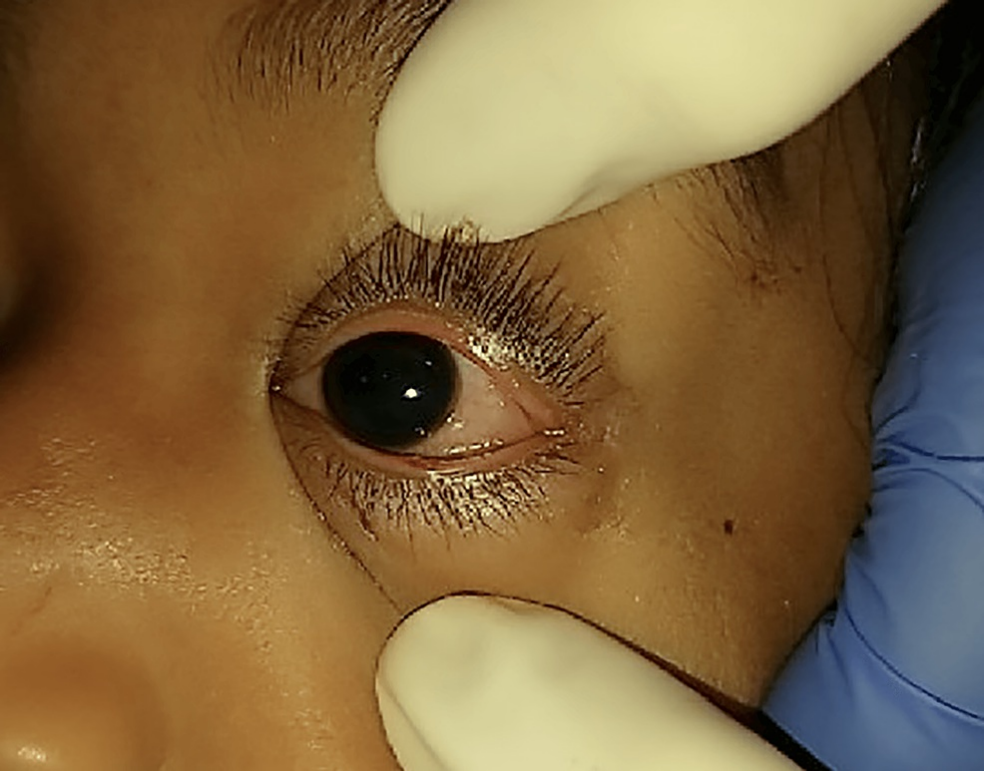
Left eye after successfully attempting the novel clamp and retract technique.

The patient was brought into the procedure room, where general anesthesia was introduced uneventfully. The patient’s left eye was prepped with ophthalmic betadine and draped in the usual sterile ophthalmic fashion. The left lower lid was noted to have a large, single-barbed fishhook passing full thickness, sparing the globe. With corneal protection, an attempt was made to cut the fishhook barb with wire cutters following the advance, and the cut removal technique, however, was unsuccessful. Due to the degree of force required to attempt this cut, it was deemed unsafe to continue. Instead, Kelly forceps were used to clamp and flatten the barb against the tip of the fishhook, creating a smooth surface against the fishhook. The fishhook was then retracted through the entry wound without resistance (Figure 2).

**Figure 3:**
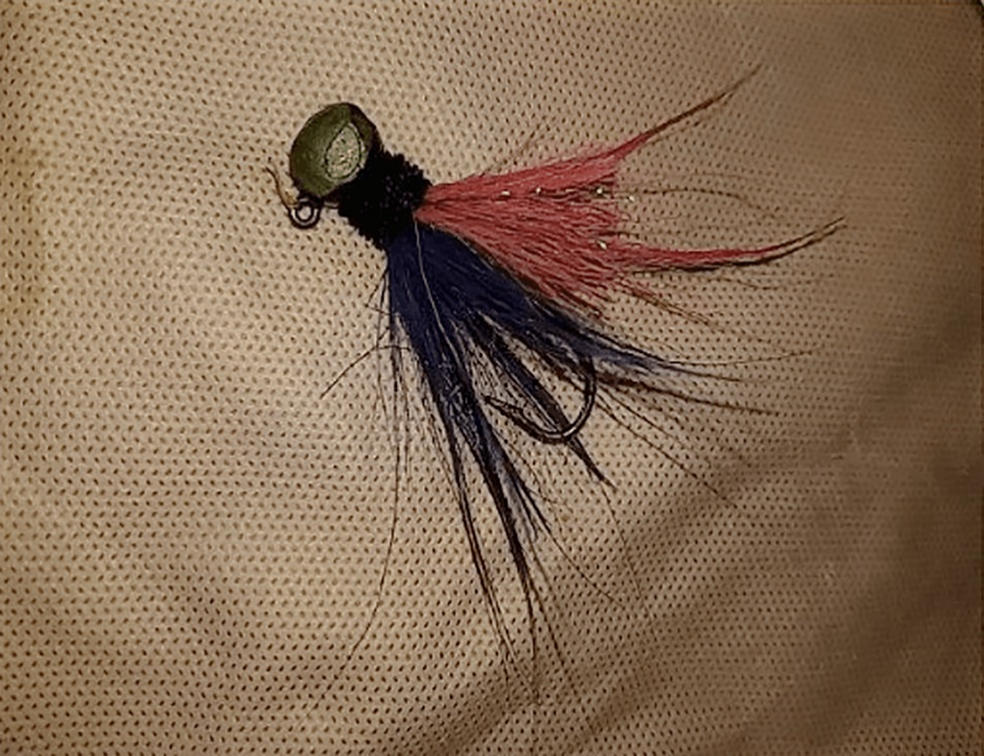
The single-barbed fishhook after successful operative removal.

The wound and eye were again thoroughly flushed with sterile irrigating and povidone-iodine solutions. After surgery, the fishhook was sent for culture (Figure 3). A dilated eye exam was performed without other further injuries. Tobramycin and dexamethasone ointment were applied to the left lower lid wound and the left lower fornix. The patient was admitted to medicine service and discharged the same day with a one-week follow-up scheduled with a pediatric ophthalmologist.

No complications resulted from the surgery. On follow-up examination of both eyes, the patient’s vision returned to baseline at 20/20 in each eye and was only significant for a 1.5mm well-approximated wound at the left external lower lid, connecting full thickness to a 1mm wound just internal to the lid margin. The left lower palpebral conjunctiva had 1+ injection, and the bulbar conjunctiva had 1+ injection inferiorly but was otherwise white, quiet, intact, and Seidel negative. The cornea had no evidence of abrasion or laceration, and the anterior chamber was deep and quiet. The iris was grossly normal, and the lenses and vitreous were clear. Dilated fundus with macula and periphery were noted to be normal.

## Discussion

Several other techniques with good surgical outcomes have been documented in the literature, but they require extra incisions, instruments, and operating time [1,2]. The vertical eyelid splitting technique is useful when there is a risk of globe penetration, and further ocular damage is inevitable due to the low visibility of the barb [3]. In cases not involving the globe, a full or partial vertical eyelid incision is made from the margin of the eyelid, connecting vertically to the fishhook. The wound can be later closed with a vertical suture. This technique was not attempted in our patient due to the hook’s barb being fully exposed. 

The retrograde technique best suits barbless or small and superficially placed barbed hooks [4]. The shank of the barb is grasped while a controlled force is placed toward the eye of the hook. Downward pressure is subsequently applied to disengage the barb from surrounding tissue, and the shank can be removed retrograde from the entry wound. While this technique may be modified by extending the entry wound by excision, this maneuver was not attempted to preserve eyelid integrity [5,6].

The needle cover technique has been demonstrated most helpful in removing fishhooks of the posterior segment of the eye [7]. For extra-ocular involvement, this technique can be used for superficial injuries when the hook shank is covered and single-barbed [3]. A needle, approximately 18 gauge or larger, is advanced into the entry wound, where the tip of the barb can meet the needle lumen. After the barb is engaged inside the lumen, the fishhook can be carefully retracted. 

The advance and cut technique is widely used for extra-ocular fishhook injuries [1]. The shank of a single-barbed hook is grasped firmly with surgical clamps, and the point of the barb is guided superficially to create a new wound opening. The barb is then transected between the bend of the shank and the barb’s edge, followed by removal. In our case, this was attempted with wire cutters. However, it was unsuccessful due to the degree of force required to attempt the cut. Instead, Kelly forceps were used to clamp and flatten the barb against the tip of the fishhook, creating a smooth surface to allow for retrograde removal through the wound entrance. 

When determining the appropriate removal technique, it is essential to consider extra- and intraocular structures involved and the type of fishhook in question. It is critical to rule out the possibility of a global injury, as this would be the most severe of cases and require immediate surgical attention by an ophthalmologist [8]. In determining the injury depth and structure involvement, ocular ultrasound, computed tomography, and x-ray have been helpful in the literature [4,9]. 

Determining whether the hook has shanks and, if so, how many is essential. In cases where the involved hook point is not visible but has exposed shanks, the non-involved shanks may be used as a reference to help in identification. This is possible because fishhook shanks are commonly uniform on individual lures. The uninvolved shanks should then be covered with tape to prevent further injury during retraction [10]. If the fishhook has only one shank, knowing the fish species involved in the event may help identify the type of lure. For example, some states only allow a single-point barbless hook for sturgeon and salmon.

## Conclusions

This is the first case reported using Kelly forceps to clamp and flatten the fishhook barb to allow retrograde removal through the eyelid wound entrance. No suture was needed for this removal, and our patient regained baseline vision without ocular complications. This case may contribute to the literature on an additional successful way of a common injury seen in ophthalmology.
